# A biofabricated 3D cancer-stroma tumor microenvironment model

**DOI:** 10.1088/1758-5090/ae0a82

**Published:** 2025-10-07

**Authors:** Sara Romanazzo, Peilin Tian, Gagan K Jalandhra, Riddhesh B Doshi, J Justin Gooding, Kristopher A Kilian

**Affiliations:** 1 School of Chemistry, UNSW, Sydney, Australia; 2 Australian Centre for NanoMedicine, UNSW, Sydney, Australia; 3 School of Materials Science and Engineering, UNSW, Sydney, Australia

**Keywords:** breast cancer, stem cells, cancer associated fibroblasts, 3D bioprinted models

## Abstract

Breast cancer progression is a consequence of intricate dynamics between cells and their matrix in the tumor microenvironment. However, most *in vitro* models are not amenable to studying the behavior of multiple cell types within a defined matrix architecture. In this study, we demonstrate a microporous matrix where breast cancer cells and adipose derived stromal cells are integrated to evaluate crosstalk between matrix parameters and heterotypic cell populations. To do this, we leveraged two biofabrication techniques, granular suspension matrices and drop-on-demand bioprinting, to deposit multiple cell types in a reproducible format amenable to high-throughput screening. 3D gelatin-methacryloyl microgels were used to create a yield stress granular suspension bath with tunable interstitial volume to mimic the porosity and densities of healthy and fibrotic microenvironments. Invasive and non-invasive breast cancer cells (MCF-7 and MDA-MB-231) were bioprinted at the interface of the ADSC-laden granular matrix to probe invasive processes and heterotypic crosstalk as a tumor–stroma model. We focused on cancer cell migration through model fibrotic tissue and ADSC transformations into cancer associated fibroblasts. *α*-smooth muscle actin expression indicated that the high density microgel matrices are more conducive to ADSC-CAF transformations, which in turn influenced the expression of molecular markers associated with tumorigenicity and chemoresistance in the resident cancer cells. Treatment with doxorubicin supported increased tumorigenicity in the co-cultures. Together, this work demonstrates how defined microengineered matrices can serve as platforms to evaluate cell behavior, with scope for translation to *in vitro* assays for biological discovery and drug development.

## 1. Introduction

Breast cancer is profoundly influenced by the tumor microenvironment, where cancer cells closely interact with various non-cancerous cell types, shaping disease progression and therapeutic outcomes [1, 2]. The crosstalk between microenvironment components and cancer cells influences changes in both cancer and non-cancer phenotypes [3]. Among the cellular components of the tumor stroma, cancer-associated fibroblasts (CAFs) are known to be crucial players, contributing to excessive extracellular matrix (ECM) production and deposition, and to promote cancer cell adhesion and migration, disrupting the normal tissue homeostasis. This phenomenon, known as desmoplasia, is particularly common in breast and pancreatic cancers [4]. Within CAFs, myofibroblasts are largely present in breast cancer tissues, and known to derive from different sources of healthy stromal cells, such as resident fibroblasts, bone marrow derived stromal cells, and adipose-derived stem cells (ADSCs). CAFs that originate from myofibroblast transitions, result in high contractility and the expression of markers like *α*-smooth muscle actin (*α*-SMA) [5].

In the last decade, many studies highlighted the importance of ADSCs in contributing to breast cancer progression. While some studies suggest pro-tumorigenic effects, such as promoting invasion and angiogenesis through paracrine secretion [6, 7], others report anti-tumorigenic activities, by inducing apoptosis in cancer cells [8, 9]. These contrasting findings highlight the need for a better understanding of the factors that govern ADSC behavior within the tumor microenvironment and their potential differentiation into CAF-like cells.

While emulating the natural architecture of living matrices in the laboratory is challenging, advances in biofabrication of three-dimensional (3D) tissue analogues have enabled the development of *in vitro* disease models [10, 11]. Among these technologies, suspension bioprinting has emerged as a promising approach for constructing physiologically and pathologically relevant 3D tissue models [12]. The support bath in these techniques is most often a jammed suspension of microscale hydrogels, providing a yield-stress fluid for deposition of cells and materials, thereby circumventing the requirement for high viscosity inks. This format allows for precise spatial control over matrix composition and cell deposition, which can be used to closely emulate the organization of cells and materials in native tissue [13–15]. Furthermore, varying the composition of the suspension solution can be used to change the interstitial space surrounding the microgels, which effectively changes the intervening porosity. In this context, biological tissue can be viewed as a porous matrix filled with interstitial fluid, while the tumor microenvironment, being less permissive, has a different permeability from the healthy tissue [13, 16, 17].

Microgels have been increasingly used to recreate tumor microenvironments and dissect cancer–stroma and immune cell interaction. For instance, guest–host interlinked PEG-MAL granular hydrogels have been applied to engineer cellular niches for cancer cell studies [18], 3D bioprinted granular hydrogel system has elucidated spatiotemporal T cell dynamics in immunotherapy models [19], and microporogen-structured collagen microgels have enabled embedded bioprinting of tumor constructs to assess immune infiltration and therapeutic responses [20]. Zheng *et al* incorporated polylactic acid into alginate microgels to achieve an extended stiffness range without altering pore size, creating a biomimetic microenvironment suited for *in vitro* tumor analysis [21]

Previously we demonstrated the use of extrusion-based printers to deposit high density cell inks within suspensions of microgels as a means to fabricate 3D cancer models [22]. This approach is useful for creating spatially organized cell populations but suffers from feature uniformity and reproducibility issues and is challenging to adapt to high throughput cell cultures [23–25]. In this study, we adapted our granular hydrogel platform [22, 26–28] for integration with a commercially available drop-on-demand bioprinter (Rastrum™, Inventia Life Sciences), to create a 3D microenvironment with tunable mechanics and matrix density that would allow to study cell-cell and cell-matrix interactions. By manipulating the properties of the microscale hydrogels (herein referred to as ‘microgels’), we replicated key biophysical parameters, such as matrix density, stiffness, and structural organization, found in healthy and cancerous breast tissues, to investigate bi-directional cross talk between tumor cells and ADSCs. Specifically, we aimed to elucidate the role of breast cancer cells in guiding ADSC transformation into CAFs, and to identify the biophysical and biochemical factors that influence this process. The insights gained from this work will not only enhance our understanding of the tumor microenvironment but also offer new opportunities for the development of therapies aimed at targeting the stromal compartment in breast cancer.

## 2. Materials and methods

### 2.1. Cell culture

Human ADSCs, purchased from ATCC (Catalogue number PCS-500-011), were cultured in low-glucose Dulbecco’s modified Eagle’s medium (DMEM, Thermo Fisher Scientific), 10% fetal bovine serum (FBS, Bovogen) and 1% penicillin/streptomycin (P/S, Invitrogen). Cells were passaged at 70% confluence using 0.05% trypsin-EDTA (Thermo Fisher Scientific) and used for experiments between passage 3–5. Human breast cancer cell line MCF-7 was purchased from ATCC (Catalogue number HTB-22). The breast cancer cell line MDA-MB-231, purchased from ATCC (Catalogue number HTB-26), was transduced with a luciferase vector encoding EGFP (figure S1) and exhibited stable EGFP expression throughout the entire cell body following integration [29]. These cells were kindly provided by Prof. Christine Chaffer from the Garvan Institute of Medical Research. Both MCF7 and MDA-MB-231 GFP were cultured in high-glucose DMEM with 10% FBS and 1% P/S. We will hereafter refer to such medium as complete medium. When ADSCs and MCF-7 or ADSCs and MDA-MB-231 were co-cultured, complete medium was used.

### 2.2. Material preparation and characterization

#### 2.2.1. Gelatin methacryloyl (GelMA) synthesis and microgel fabrication

GelMA was synthesized as previously shown [26, 30]. Briefly, type A gelatin from porcine skin, gel strength 300 (Sigma-Aldrich) was dissolved to 10 w/v% in 1x phosphate buffer solution (PBS) at pH 7.4 at 50 °C under stirring. Methacrylic anhydride was added (5 v/w% of the total mixture) and mixed for 90 min at 50 °C. The solution was then diluted two-fold with PBS and centrifuged at 3000 rcf (3 min) to pellet and remove excess methacrylic anhydride. The obtained supernatant was dialyzed with a 14 kDa cutoff at 40 °C for 5–7 d against distilled water, and by daily changing water. The GelMA solution was then lyophilized for 5 d before long term storage at −20 °C. When needed to fabricate GelMA microgels, lyophilized GelMA was reconstituted in PBS to a final concentration of 10 w/v%. An oil-emulsion method was used to generate microgels of the average size of 100 *μ*m in a natural oil bath (Sunflower oil, Community Co.). The obtained microgels were stored in 100% acetone solution at room temperature (RT) until the day of the experiment. For microgel hydration, the acetone was removed via evaporation, and appropriate volumes of DMEM, 1% w/v GelMA solution in DMEM and lithium phenyl-2,4,6-trimethylbenzoylphosphinate (LAP) photo-initiator were added and incubated overnight at RT [28], to ensure homogeneous hydration of the microgels in the different groups. DMEM facilitated microgel rehydration, while 1 w/v% GelMA served as a filler to control the spacing between microgels, thereby adjusting the overall packing of the hydrogels.

Each of the microgel hydrogel formulation was loaded at the volume of 100 *μ*l per well in a 96 well-plate, photo-crosslinked with a torch emitting on the visible light range of 405 nm and imaged on a confocal microscope (Zeiss LSM800) using a 488 nm laser, at which microgels show autofluorescent signal. The obtained microgel hydrogels were called low to high microgel packing density based on the increasing amounts of DMEM and 1 w/v% GelMA (table 1). Compactness of each microgel hydrogel was quantified from 3D confocal renderings by calculating the percentage of construct volume occupied by microgels (microgel volume ÷ total volume × 100). Imaris software (version 9.9.1) was used to assemble the images and perform the volume calculations.

**Table 1.** Microgel hydrogels formulation for each low, medium and high microgel packing groups (100 mg microgels each).

	DMEM (ml)	1 w/v% GelMA (ml)	2.5 w% LAP (ml)
Low	5.69	1.27	0.140
Medium	0.84	3.76	0.094
High	1.80	0	0.037

#### 2.2.2. Rheological analysis

Crosslinked microgel hydrogels were also characterized for their mechanical properties by using an Anton Parr Modular Compact Rheometer (MCR 302e) equipped with a 25 mm parallel plate and quartz stage. Pre-crosslinked microgel hydrogels were placed on the stage and the gap was set to 1 mm. Strain sweep test was performed using a log ramp rate from 0.02% shear strain up to 200% at 1 Hz frequency over 8 min.

### 2.3. 3D bioprinting

3D cell culture models were bioprinted using a custom-designed drop-on-demand 3D bioprinter (manufactured by Inventia Life Science). The bioprinter incorporates a flyby printhead, allowing high-throughput printing in 96-well and 384 well-plates. The Rastrum printer was previously described in detail by Utama *et al* [23]. A high-density solution of cells, containing either MCF-7 or MDA-MB-231 at a concentration of 10^7^ cells ml^−1^, was primed into the nozzle from a bioprinting cartridge. Five droplets per well of around 19 nl each, were printed for each construct at a pressure of 30 kPa, in either a 96 or 384 well plate, depending on the purpose of the experiment, where microgels solutions were pre-loaded before cell printing. Other bioprinting parameters were defined in a custom-made software from Inventia Life Science. At the end of the printing process, the entire 96 or 384 well-plate, was exposed to UV light (405 nm) for 90 sec, to allow photo-crosslinking of the microgel hydrogels or bulk hydrogels. The plate was then incubated at 37 °C and 5% CO_2_ for 5 min, after which 100–200 *μ*l of culture media was added to each well and placed back in the incubator for the required time of each experiment. The experimental setup, including the cancer cell lines, microgel packing conditions, and CAF activation groups, is illustrated in figure S2.

### 2.4. Immunohistochemistry and confocal imaging analysis

Samples were fixed in 4% paraformaldehyde (Bio-Strategy Ltd) solution overnight at 4 °C at the desired time point. Following, cell membranes were permeabilized by incubation in 0.1% Triton X-100 for 3 h. Primary antibodies were diluted 1% bovine serum albumin (BSA) (1:100; Invitrogen) and incubated for 24 h at 4 °C. Appropriate secondary antibodies were diluted in 1% BSA (1:200) and incubated for 24 h at 4 °C. Nuclei were counterstained with DAPI (Invitrogen). Samples were incubated in CUBIC-2 solution for 24 h at room temperature before being imaged with Zeiss LSM 800 confocal microscope. Images analyses were performed using Imaris software (x64 9.9.1).

### 2.5. Total RNA isolation and quantitative reverse transcription polymerase chain reaction (qRT-PCR) for miRNA expression analysis

Conditioned medium from each group was collected and used to analyze their miRNA content. Briefly, total RNA was isolated with miRNeasy Micro Kit (Qiagen, Catalog number 217084), which was subsequently retro-transcribed into cDNA with miRCURY LNA RT Kit (Qiagen, catalog number 339340) according to the manufacturer’s instructions. Real time qRT‐PCR was then performed using Quantstudio 12 K Flex Real-Time PCR system (Applied Biosystems). The thermocycling conditions were 95 °C for 2 min, followed by 40 cycles of 95 °C for 15 sec and 60 °C for 1 min. Normalization of the data was performed using two micro-RNAs (miRNAs) recognized as being stable US6 and SNORD44. The primers used in this study are listed in table 2. The specificity of the SYBR PCR signal was confirmed by melt curve analysis. Ct values were transformed into relative quantification data using the 2^−ΔΔCt^ method, and data were normalized to the average of US6 and SNORD44 expression.

**Table 2.** List of primers used to detect miRNA expression through qRT-PCR. miRNAs are listed based on their names on the miRbase and their corresponding product code from Qiagen.

miRNA name	Product code
SNORD44 (used as reference)	YP00203902
U6 snRNA (used as reference)	YP02119464
hsa-miR-125b-5p	YP00205713
miR-146a-5p	YP00204688
hsa-miR-125b-1-3p	YP00204400
hsa-miR-222-3p	YP00204551

### 2.6. Proteomic analysis of cell secretome

Conditioned medium from each group was collected and used to analyze their secretome profile through LCS-MS, as previously established by the Bioanalytical Mass Spectrometry Facility the Mark Wainwright Analytical Centre at UNSW [31]. Peak lists were generated using Mascot Distiller (Matrix Science) and submitted to the database search program Mascot (version 2.8.3, Matrix Science). Search parameters were: precursor tolerance 4 ppm and product ion tolerances ± 0.05 Da; Met(O) carboxyamidomethyl-Cys specified as variable modification, enzyme specificity was trypsin, 1 missed cleavage was possible, and the UniProt database searched. Scaffold 5 software (version 5.3.3) was used for data analysis.

### 2.7. Drug response study

High-throughput 3D bioprinting in 384 well-plate was performed to allow a drug response study without using too much material. 3 d post-print, samples were exposed to increasing concentrations of doxorubicin (up to 100 *μ*M), a well-known chemotherapeutic drug used in patients with aggressive breast cancers. Doxorubicin stock solution was prepared by dissolving doxorubicin hydrochloride (Sigma-Aldrich) in PBS at 5 mg ml^−1^ concentration. Working solutions were prepared by mixing doxorubicin stock solution with complete medium. Ethanol 30% diluted in complete medium was used as negative control. A 50 *μ*l aliquot of drug supplemented medium was replaced after 2 d of culture. At day 5, doxorubicin effect was assessed by using CellTiter-Glo® (Promega Corporation) following the manufacturer instructions. Dose response curve and IC_50_ calculation for each experiment were generated in GraphPad Prism software (version 10.3.1.). In order to detect doxorubicin diffusion in the matrices, increasing concentrations of the drug (10 and 100 *μ*M) were added to the constructs through media, and its presence was detected by confocal microscopy with laser settings at 470 nm excitation and 560 nm emission.

### 2.8. Statistical analysis

Results are expressed as mean $ \pm $ standard deviation of mean of n $ \ge $ 3 independent experiments. Statistical analyses were performed using GraphPad Prism (version 10.3.1.) software. One- or two-way analysis of variance (ANOVA) were used for ANOVA to compare between groups.

## 3. Results and discussion

### 3.1. Tuning microgel properties to mimic normal and cancerous breast tissue *in vitro*

**Figure 1.**
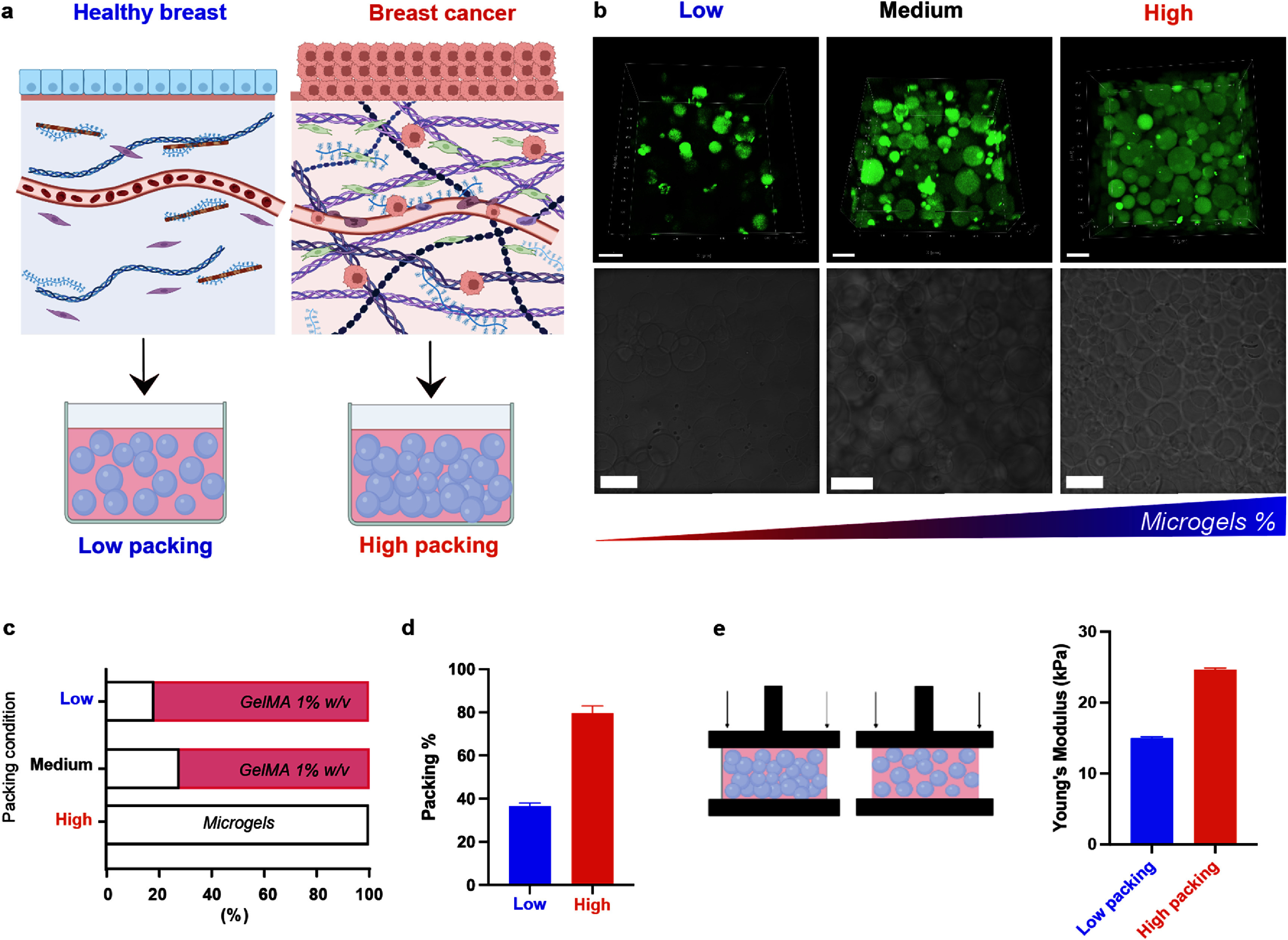
Tuning microgel suspension properties to mimic breast ECM *in vitro*. (a): schematic representation of proposed rational for using microgels to mimic healthy (left) and cancerous (right) breast tissue; (b): representative confocal (top) and brightfield (bottom) images of increasing packing of microgels’ %; (c): microgel content *vs* interstitial GelMA (1% w/v); (d): porosity of microgel composites quantified through analysis of void space; (e): schematic of mechanical testing (compression) of crosslinked microgel matrices and corresponding Young’s Modulus (*n* = 5). Scale bars = 100 *μ*m.

Breast tissue displays heterogeneity with soft and stiff regions and intervening fluid phases [32]. ECM density is in fact a key feature distinguishing healthy and cancerous breast tissues. In cancerous tissues, ECM density increases significantly compared to healthy tissue. To mimic the matrix heterogeneity of native healthy and cancerous breast tissues, we used granular suspensions of GelMA microgels with different packing densities to tune individual microgels and bulk mechanical properties [26–28]. Modulating the packing of GelMA microgels with a soft interstitial GelMA phase to create space between the spheres, allowed us to generate a range of tissue mimics (figure 1(c)). Microgel hydrogels compositions are detailed in table 1 in the materials and methods section. As illustrated in figure 1(a), healthy breast tissue is generally characterized by a soft and low dense matrix where cell interaction and signaling are regulated and occur in a highly controlled manner to support physiological functions. On the contrary, breast cancer tissue is defined by higher matrix density, due to the increased secretion and crosslinking of ECM components, such as collagen fibers and proteoglycans. Women with high-density breast tissue are 4–6 times more likely to develop malignant tumor than women with less dense tissue [33]. We translated this into an *in vitro* model by preparing composite hydrogel solutions containing microgels at a different distance from each other (packing), designed to mimic healthy and cancerous breast tissue environments. The tightly packed condition contained a higher proportion of microgels with limited space in between, so that we could mimic the dense matrix of cancerous tissue and was confirmed by brightfield and confocal imaging (figure 1(b), right), while the hydrogel with low packing of microgels condition presented more dispersed microgel distribution (figure 1(b), left). In order to achieve a structural condition where microgels were highly dispersed, we filled the space between microgels with a bulk GelMA 1% w/v hydrogel (figure 1(c)). Adding GelMA to these constructs leads to differences in microgel packing density but also changes the interstitial content, likely introducing some nanoscale mesh in the pore space. However, we have previously shown that the addition of a low GelMA content (1% w/v) to the interstitial space does not significantly affect cell migration in these microporous scaffolds [22, 26]. This suggests that while the interstitial GelMA will also affect cell movement, some microscale pore space remains accessible to allow migration through the material. Nevertheless, it is important to note that we are changing both microgel spacing and void content in the cancerous mimic. Quantification of microgels percentage confirmed the differences in composition, with 35 and 80% packing conditions, for healthy and cancerous tissues, respectively (figure 1(d)). To further characterize microgel hydrogel conditions, we measured their mechanical properties by determining the Young’s modulus of the hydrogels through compression testing. The cancer hydrogel mimic resulted in a significantly higher Young’s modulus (25 kPa) compared to the healthy hydrogel mimic (10 kPa) (figure 1(e)). The range of Young’s moduli found in literature for breast cancer and healthy breast tissues ranges between 9–12 kPa for the cancer tissue, and between 0.5–1 kPa for the healthy tissue [34]. It should be noted that these heterogeneous hydrogels will have phases with variable moduli; for instance, while the average modulus of the healthy mimic is 10 kPa, the high content interstitial GelMA phase (1% w/v) is 64 Pa while the stiffness of the microgel phase is 34.9 kPa. Therefore, the modulus experienced by the cells within the material will vary broadly, akin to what cells might experience in real tissue. The absolute stiffness values of our healthy and cancerous constructs exceed the physiological ranges reported for native tissues, in part due to materials limitations and the need for stable scaffolding for long term cultures. Nevertheless, the differences in mechanics and architecture elicit variations in cell behavior that align with native activities. To assess the effect of microgels on cellular mobility and distribution, we compared high cell suspensions printed in both microgel and bulk hydrogel systems (figure S3(a)). Using the Rastrum™ bioprinter, a high-density cell solution was 3D printed on the surface of either a microgel suspension or continuous GelMA hydrogels, with the same GelMA weight content. After 3 d of culture, cells printed in microgel suspensions showed more uniform infiltration in all directions, as evident from the 3D confocal images, whereas those printed in a uniform GelMA hydrogel of equivalent w/v% were not able to migrate within the matrix and remained clustered near the original printing site (figure S3(b)). Cells in the bulk hydrogel also did not penetrate into the matrix, travelling no more than 100 *μ*m from the top surface of the gel, while cells in the microgel system, were able to penetrate up to 300 *μ*m in 3 d, confirming a more cell-permissive environment compared to uniform hydrogels (figure S3(c)). These results demonstrate how microgel suspensions can be used to study cell infiltration.

### 3.2. Breast cancer cells respond to different matrix microenvironments

Here we evaluate the use of the Rastrum™ drop-on-demand bioprinter to deposit high density cell populations at the surface of the microgel suspensions, where printing parameters can be varied to modify droplet size, cell density and 3D penetration, towards using this platform for high throughput biology.

**Figure 2.**
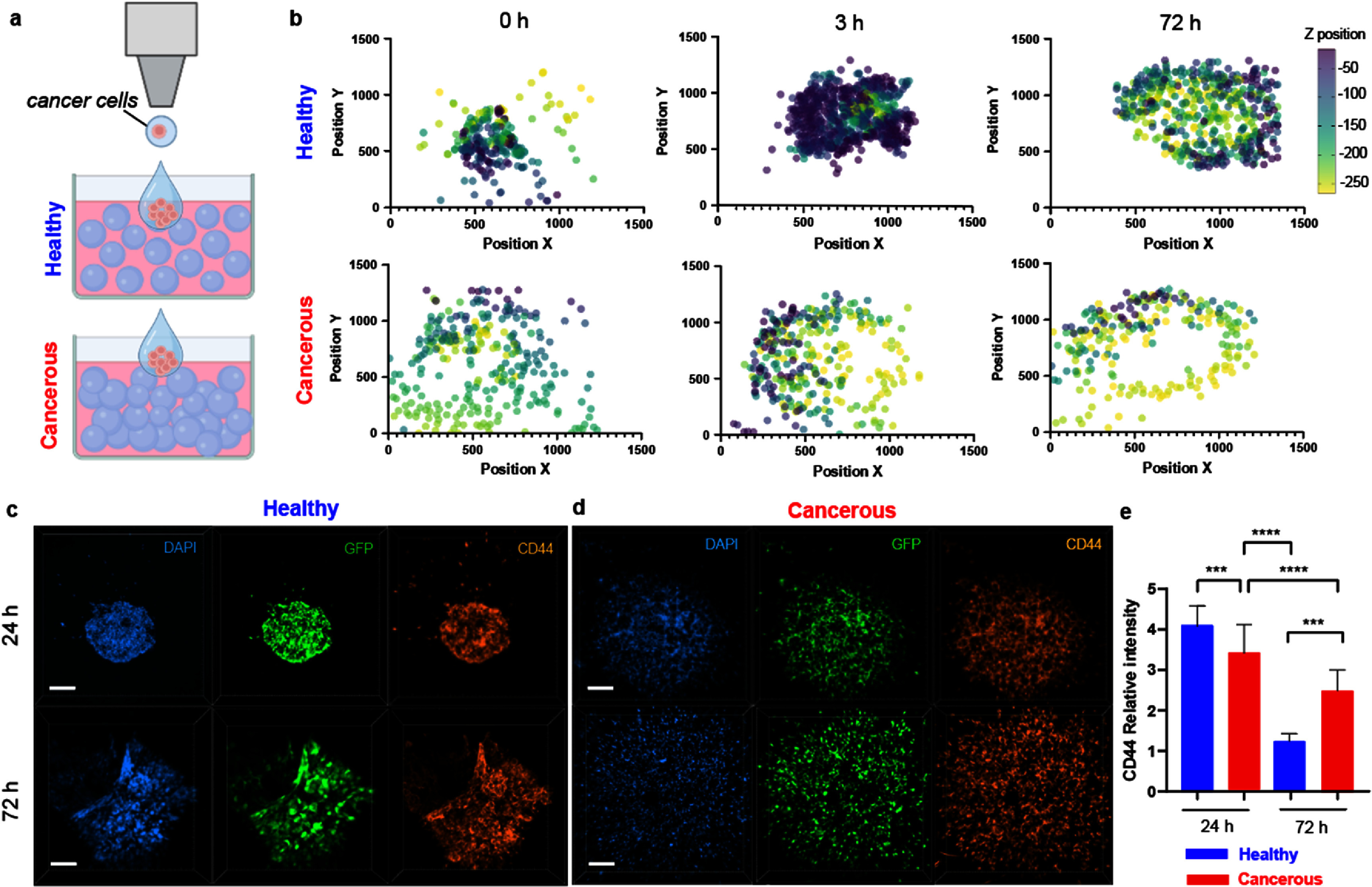
Aggressive breast cancer cells respond to different microgel packing content. (a): schematic representation of the experimental setup: cancer cells were printed using a drop-on-demand printer into either low-density (healthy) or high-density (cancerous) microgel hydrogels; (b): 3D scatter plots of MDA-MB-231 breast cancer cells x-y-z positions in microgel hydrogels mimicking healthy (top) and cancerous (bottom) tissues right after printing (0 h) and after 24 h and 72 h; (c): immunofluorescence images of breast cancer cells in healthy mimic and (d): in cancerous tissue mimic, stained for CD44 (orange); nuclei were counterstained with DAPI (blue); (e): quantification of CD44 expression levels in both healthy and cancerous groups; ***p < 0.001, **** p < 0.0001; Scale bars =200 *μ*m.

To investigate the impact of varying microgel density content on breast cancer cell behavior, we dispensed and then cultured aggressive breast cancer cells, GFP labeled MDA-MB-231, in microgel hydrogels with either low or high microgel packing content (figure 2(a)). We monitored cell spatial distribution, and expression of CD44 over time. CD44 expression has been associated with tumor severity and tumor recurrence in patients with breast cancer and has been also correlated to metastasis [35] and drug resistance [36].

3D image reconstructions and the 3D scatter plot revealed significant differences in cell infiltration between healthy mimic and cancerous mimic hydrogels over 72 h from printing (figure 2(b)). Moreover, in the healthy mimic, cancer cells remained clustered with limited spatial dispersion and infiltration over time, while in the cancerous mimic, cells showed a dispersed and deeper migration into the matrix (figure 2(b)). Interestingly, in the healthy mimic, cell infiltration initially reached a 300 *μ*m depth in *Z* direction but gradually migrated toward the hydrogel surface (−100 *μ*m). This is attributed to the initial cell infiltration due to the pressure applied during printing, which allowed cancer cells to penetrate further in the low-density healthy mimic where the void space is higher compared to high density cancerous mimic. Cell migration results correlated well with CD44 expression, with overall CD44 levels being higher in cancerous mimic (figures 2(d) and (e)) when compared to those in the healthy mimic (figures 2(c) and (e)). Similar results were observed when the non-aggressive breast cancer cells, MCF-7, were printed in the same conditions (figure S4).

Cells cultured in the dense matrix mimicking cancerous tissue, show adhesion and migration along the microgel surfaces, which also corresponded with increased expression of CD44 (figure S5, top), whereas in the healthy mimic they remained rounded and clustered (figure S5, bottom). These observations align with previous findings where strong cell-matrix adhesions have shown to enhance invasive traits and downstream signaling [37–39]. Although we did not isolate stiffness as an independent variable, we acknowledge that matrix stiffness can modulate cell adhesion, contractility, and migration, as demonstrated in previous mechanobiology studies [39, 40]. On the contrary, cells embedded in the healthy mimic faced adhesion limitations due to the interstitial GelMA 1% phase. Together, these results show how drop-on-demand printing is amenable to granular materials and that varying matrix density and interstitial content can be used to mimic invasive pathways to study cancer cell migration.

### 3.3. Both cells and matrix contribute to ADSC transitions to CAF phenotypes

**Figure 3.**
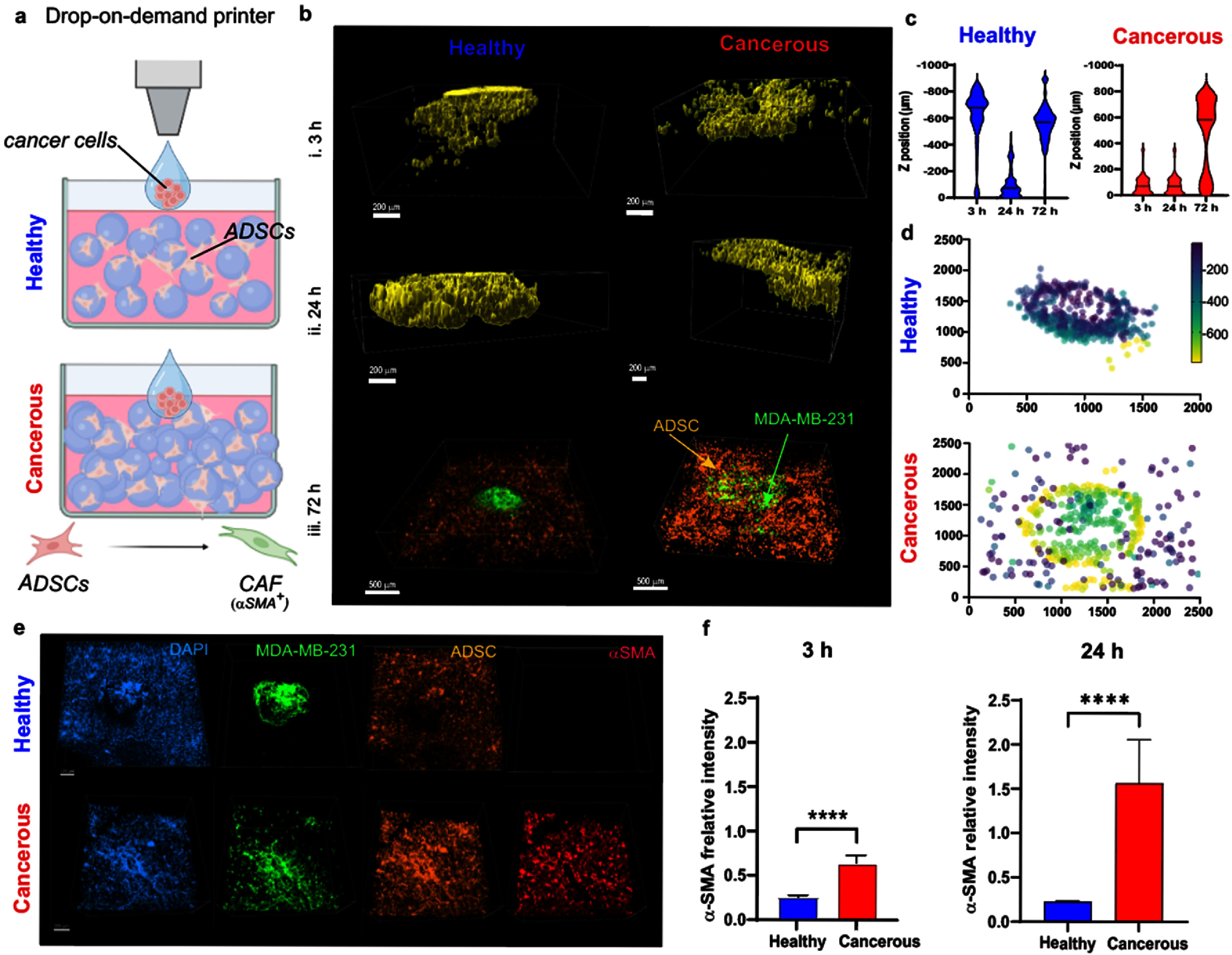
ADSCs transition to a CAF phenotype when in co-culture with MDA-MB-231 in cancerous mimic matrices. (a): Schematic representation of the experimental setup: cancer cells were printed using a drop-on-demand printer into either low or high microgels content where ADSCs were introduced and their transformation into cancer-associated fibroblasts (CAFs) expressing *α*-SMA was evaluated; (b): 3D reconstruction images showing the spatial positioning of ADSCs within the tissue mimics at different time points (3 (i), 24 (ii), and 72(iii) h) following the printing; (c): violin plots quantifying the Z position (depth) of ADSCs within the low (healthy) and high (cancerous) microgel density conditions at different time points; (d): 3D scatter plots of cancer cell x-y-z positions at 72 h; (e): representative confocal microscope images from healthy and cancerous tissue mimics at 24 h post-printing, showing CAF marker *α*-SMA (red), nuclei (DAPI), MDA-MB-231 cancer cells (green) and ADSCs (orange), scale bars: 100 *μ*m; (f): quantification of α-SMA expression at 3 h (left) and 24 h (right) post-print; **** p < 0.0001.

To explore the role of matrix density in ADSCs transformations to CAFs phenotypes, ADSCs were co-cultured with aggressive breast cancer cells (MDA-MB-231) in healthy and cancerous tissue mimics (figure 3(a)). Initially, we evaluated ADSCs behavior in mimics alone, to determine whether mechanophysical differences themselves influenced their differentiation into CAFs. It is well-established that ADSCs are highly plastic and responsive to physical stimuli. However, ADSCs showed no positive expression for *α*SMA, a CAF marker, in either healthy or cancerous conditions when cultured alone (figure S6). We then evaluated the cancer cell penetration into the matrix in the presence of uniformly dispersed ADSCs over the period of up to 3 d. We noticed that breast cancer cells penetrated to a greater depth when presented with the cancerous mimic despite an initial resistance to infiltration, as previously observed in mono-culture (figures 3(b)–(d)). Interestingly, cancer cells showed significantly greater migration in the presence of ADSCs, suggesting a strong signaling interaction between the two cell types. This interaction reveals potential mechanisms by which ADSCs contribute to cancer cell invasion and metastasis. Notably, MDA-MB-231 cells were found at distances up to 1000 *μ*m from the printing site, in contrast to <350 *μ*m when cultured without ADSCs, suggesting the stromal cells are secreting factors to stimulate cancer cell migration. To determine whether ADSCs were differentiating into CAFs, we evaluated *α*-SMA expression through immunofluorescence staining. At 24 h, ADSCs cultured in the cancerous mimic displayed a significantly higher expression of *α*-SMA, suggesting this microenvironment is conducive to CAF transformations. In contrast, *α*-SMA expression was minimal in the healthy mimic hydrogels, where ADSCs showed little evidence of differentiation into CAFs, and only when in direct contact with cancer cells (figure 3(e)). Quantitative analysis resulted in significantly higher *α*-SMA presence in cancerous versus healthy mimics at 3 h post-print (figures 3(f) and S7(a)). The same trend persisted at 24 h (figures 3(e) and (g)).

Similar findings were observed when ADSCs were co-cultured with the non-aggressive breast cancer MCF-7 cells (figure S8), where *α*-SMA was overexpressed both at 3 h and 24 h post-print in ADSCs cultured in cancerous mimic, compared to the healthy mimic (figures S8(a), (b–i), (c–i)). Higher expression of α-SMA corresponded with higher cell volume and more elongated cell and nuclear shapes (figures S8, (b–ii), (b–iii), (c–ii), (c–iii)), a phenotypic characteristic of myofibroblastic CAF subpopulations [41]. This trend was also observed in co-cultures of ADSCs and MDA-MB-231 (figure S7(b)). It has been previously demonstrated that during CAF transition, cells change their morphology at the nuclear and cellular level [42].

The significant differences in cancer cell migration distances in the presence and absence of ADSCs in the cancerous mimic suggest a complementary interaction between the two cell types, consistent with findings reported in the literature. It has been reported that ADSCs secrete paracrine factors, that enhance cancer cell motility or degrade the surrounding matrix, facilitating deeper infiltration [43]. Promny *et al* found that irradiated MCF-10 A, a non-malignant breast epithelial cell line, exposed to conditioned medium from ADSCs underwent an epithelial-to-mesenchymal (EMT) transition, with increased matrix secretion/deposition, suggesting a pro-tumorigenic effect of ADSCs [44]. CAFs have also been shown to induce local matrix stiffening [45, 46], which may further influence cancer cell invasion; however, investigating matrix stiffening within embedded microgels presents technical challenges and remains an avenue for future study. This co-dependence further emphasizes the importance of having a system capable of replicating tumor–stroma crosstalk *in vitro* to better analyze its role in shaping the invasive potential of cancer cells.

### 3.4. Secretory profiles align with cancer-stroma bi-directional communications

**Figure 4.**
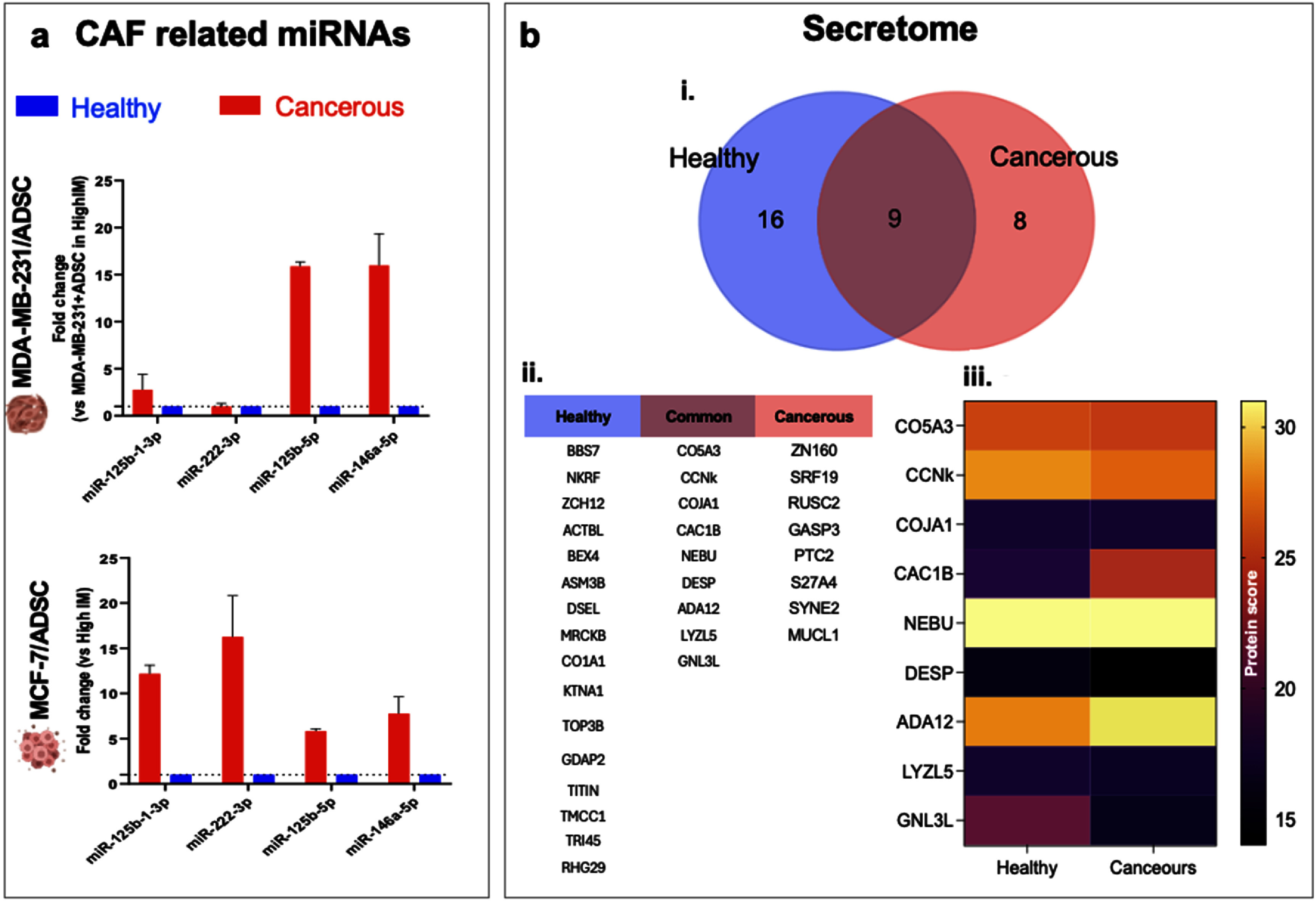
3D microgel cultures direct changes in secretome profiles. (a): CAF-related miRNA expression profiles in conditioned media from co-cultures of breast cancer cells (MDA-MB-231, top; MCF-7, bottom) with adipose-derived stem cells (ADSCs) grown in either low (Healthy) or high (Cancerous) microgel matrix conditions. (b): Secretome analysis of co-cultures under healthy and cancerous conditions; (i): venn diagram shows the number of uniquely or commonly secreted proteins in each condition; (ii): table lists identified proteins categorized as unique to healthy, cancerous, or common to both; (iii): the heatmap displays relative protein scores for a subset of selected proteins across both matrix conditions.

We next sought confirmation of the heterotypic phenotypical changes observed through confocal microscopy, by investigating the secretome profiles in order to correlate cell behavior with their secreted factors. A key messenger involved in cellular communication is the secreted regulatory molecules miRNAs. miRNAs play a crucial role in regulating gene expression at the post-transcriptional level, transiting cells and microenvironments through exo-/endo-cytosis via extracellular vesicles, impacting key processes such as proliferation, apoptosis, differentiation, and metastasis [47]. Dysregulated miRNAs expression has been implicated in breast cancer development and progression, influencing tumor growth, invasion, and tumor response to therapies [48]. Specific miRNAs are known to inhibit crucial proteins in cancer cell survival, and even the same miRNA can have both pro- or anti-tumor activity, depending on the specific target they are considered for. We focused in particular on the miRNAs associated with CAF appearance in breast cancer [49]: miR-125, miR-222 and miR-146 families. By exploring miRNA profiles, we tried to gain deeper insights into the molecular mechanisms driving ADSC activation into CAF. We analyzed conditioned media from co-cultures of aggressive (MDA-MB-231) and non-aggressive (MCF-7) breast cancer cells with ADSCs under healthy and cancerous matrix conditions (figure 4).

In the MDA-MB-231/ADSC co-culture system, miRNA analysis revealed significant upregulation of several miRNAs in ADSCs cultured in the cancerous mimic compared to those cultured in the healthy mimic hydrogels. Specifically, miR-125b-1-3p, miR-222-3p, miR-125b-5p, and miR-146a-5p were all significantly elevated in the cancerous mimic, with miR-125b-5p and miR-146a-5p showing the highest fold changes (approximately 15-20 fold). In contrast, miRNA expression in the healthy mimic remained consistently low across all targets, suggesting that culture in the dense fibrous cancerous mimic promotes the release of miRNAs associated with cancer aggressiveness and stromal remodeling (figure 4(a), top). Similarly, in the MCF-7/ADSC co-culture system, miRNA expression was significantly upregulated in the cancerous mimic compared to the healthy mimic. miR-125b-1-3p, miR-222-3p, miR-125b-5p, and miR-146a-5p exhibited increased expression, with miR-222-3p and miR-125b-1-3p showing the highest fold changes (10-15 fold) from cells cultured in the cancerous mimic (figure 4(a), bottom). As seen with the aggressive MDA-MB-231 cells, the healthy mimic fostered low expression of these miRNAs from resident cells, indicating that this microenvironment suppresses the secretion of these key regulatory miRNAs. Moreover, when comparing the secreted medium of co-cultures to monocultures of MDA-MB-231 under the cancerous and healthy conditions, the latter co-culture group had the lowest expression levels of all the analyzed miRNAs, suggesting that ADSCs may acquire anti-cancer properties when exposed to the appropriate environment, such as the healthy mimic hydrogels (figure S9). The miR-125 family has been associated with cell proliferation, invasion, differentiation, and drug resistance. In particular miR-125-b induces resistance of breast cancer cells to paclitaxel by suppressing the apoptosis, regulates EMT and induces metastasis [50]. miR-222-3p also promotes proliferation of cancer cells, EMT transition [51] and metastases [52]. MiR-146a-5p promotes cell proliferation [53], regulates CAF formation and subsequent cancer metastasis [54].

When we analyzed the conditioned medium for their protein content, we found 16 proteins uniquely secreted from cells cultured in the healthy mimic, 8 proteins exclusively secreted from cells cultured in the cancerous mimic, and 9 proteins shared between the two conditions (figure 4(b)). Proteins specific to the cancerous mimic include those involved in matrix remodeling and tumor–stroma interaction, such as MUC1L, which is associated with cancer progression [55]. In contrast, proteins unique to the healthy mimic system, such as ACTBL2 and KRTAP, may indicate a more stable or constrained environment that suppresses aggressive cancer behaviors (table S1). Proteins expressed in both systems, including cyclin K (CCNK) and guanine nucleotide-binding protein-like 3 (GNL3L) were found upregulated in the cancerous mimic relative to the healthy tissue mimic. CCNK controls cell cycle and transcriptional activities by forming complexes with cyclin-dependent kinases CDK9 and 12. CDK9 has recently emerged as a potential therapeutic target for cancer, because of their high presence in triple negative breast cancer types [56, 57]. Together, these panels confirm that culture in the cancerous mimic promotes a secretome consistent with aggressive cancer behavior and stromal remodeling, whereas the healthy tissue mimic maintains a less permissive and more stable environment. This demonstrates the interconnected roles of miRNA regulation and protein secretion in controlling tumor–stroma crosstalk under different matrix conditions.

### 3.5. Breast cancer cells exposed to Doxorubicin show signs of resistance in cancerous matrix mimics

**Figure 5.**
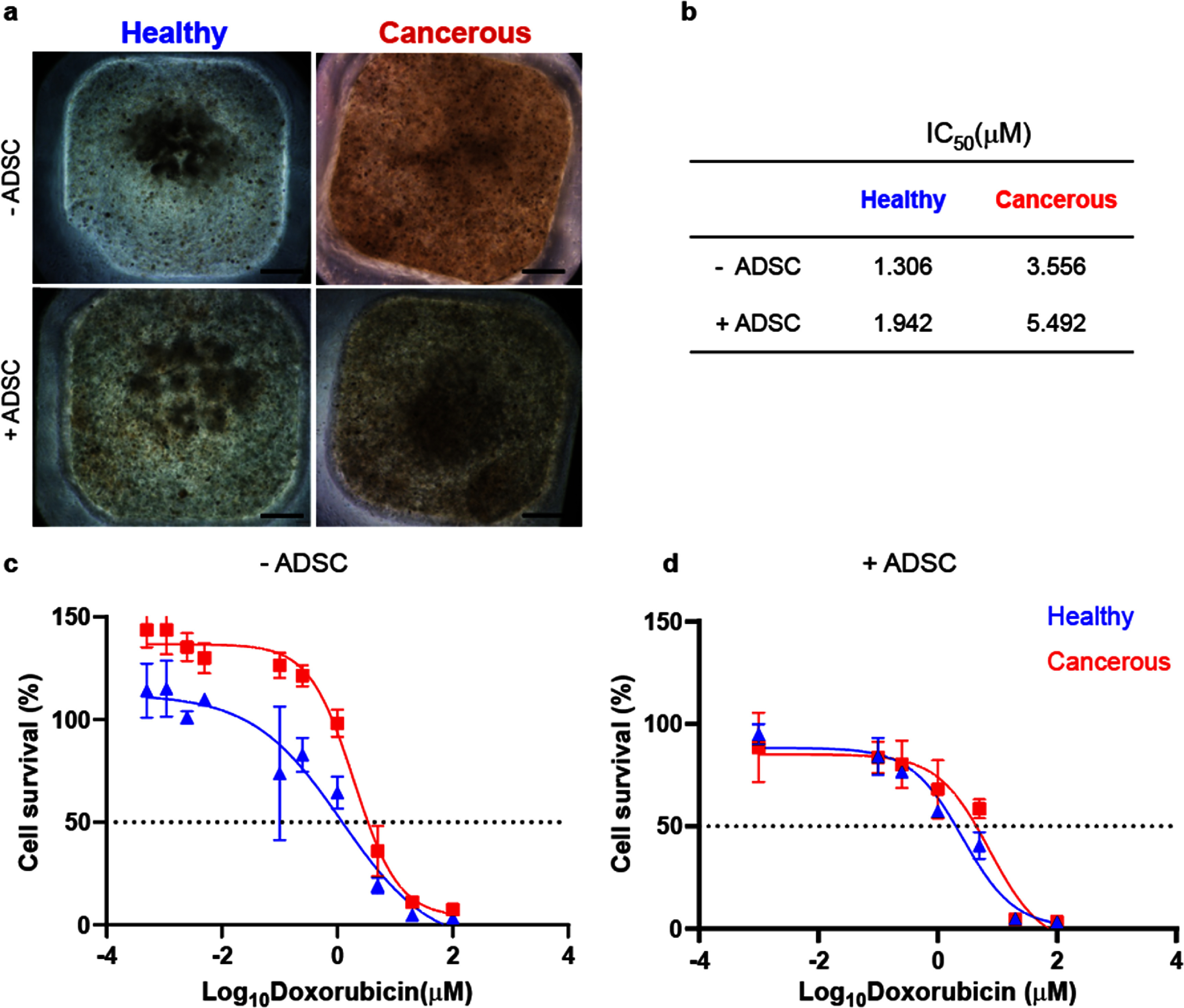
Effect of microgel density and ADSCs on breast cancer cell response to doxorubicin treatment. (a): Brightfield images of cancer cell clusters in low (Healthy) or high (Cancerous) microgel density hydrogels, in absence (top) or presence (bottom) of ADSCs; scale bars: 500 *μ*m; (b): table showing IC_50_ values extrapolated from dose-response curves in c and d, showing the effect of doxorubicin in absence (c) or presence (d) of ADSCs.

To validate our 3D model, we next tested aggressive breast cancer cell line with a commonly used chemotherapeutic drug, doxorubicin, to evaluate relationships between cancer-stroma interactions and drug sensitivity. As a first step, we scaled-down the constructs size into 384 well-plates and verified that cell behavior did not change compared to previous experiments performed in 96 well plates. The drop-on-demand printing technology allowed accurate printing on this scale, providing scope for translation to high throughput pipelines. Representative images in figure 5(a), illustrated the macroscopic morphological differences in tumor constructs under conditions of our healthy and cancerous mimics, both with and without ADSCs, with cancer cells forming a more localized cluster when cultured in the healthy mimic compared to the ones cultured in the cancerous mimic, similarly to the previously tested 96 well plate samples. Within the normal tissue groups, in the absence of ADSCs, cancer cells exhibited a defined and localized migration pattern at day 1, which over time slightly expanded (highlighted in yellow in figure S10). However, when ADSCs were added, the boundaries of the cancer cell migration became less distinct, with a more dispersed and diffuse pattern observed, particularly by day 3 (figure 5(a) + ADSC, figure S10). Results from the quantitative analysis of the cell clusters observed in healthy mimic hydrogels confirmed that the cell density was higher in the absence of ADSCs compared co-culture (figure S10, bottom). This indicates a noticeable impact of ADSCs on the reorganization and behavior of the cancer cells.

When samples were subjected to increasing concentrations of doxorubicin, the cancerous mimic cultures resulted in a higher IC_50_ value (3.556 *μ*M) compared to the healthy cultures (1.306 *μ*M), indicating that cells in the high microgel packing group were more resistant to doxorubicin treatment (figure 5(c)). When ADSCs were present (figure 5(d)), both conditions showed a shift in the IC_50_ values. The IC_50_ increased to 5.492 *μ*M and to 1.942 *μ*M for the cancerous and healthy mimic conditions respectively, suggesting that the presence of ADSCs conferred additional resistance to doxorubicin across both conditions (figure 5(b)). Overall, the results demonstrate that cancerous mimic hydrogels promote cancer aggressiveness and that the presence of ADSCs further enhances the resistance of cells to chemotherapy. To ensure that differences in treatment response were not driven by variable drug diffusion across matrices, we assessed doxorubicin penetration in both microgel constructs by exploiting the compound’s intrinsic autofluorescence. Constructs were incubated with either 10 *μ*M or 100 *μ*M doxorubicin for 24 h, followed by z-stack confocal imaging. Quantitative analysis of fluorescence intensity profiles revealed a nearly identical distribution of doxorubicin in low- and high-packing matrices at all depths examined (figure S11). These results demonstrate that drug diffusion is equivalent under both matrix conditions, indicating that the observed differences in cell viability arise from genuine variations in cell–matrix interactions and mechanotransduction rather than from altered drug penetration.

## 4. Conclusion

In this study we demonstrate a 3D biofabricated model combining tunable granular matrices with drop-on-demand bioprinting, which was able to reproducibly replicate cancer cell and ADSC co-culture to mimic key aspects of the tumor microenvironment. We demonstrated that a high density microgel culture without interstitial matrix—containing an interconnected pore network—(so-called ‘cancerous mimic’) promotes breast cancer cell invasion, stemness, and chemoresistance, while low density microgels with a soft interstitial matrix (so called ‘healthy mimic’) discourages invasive spreading and adoption of chemoresistant stem cell-like states. These model microenvironments allowed co-culture of ADSCs with breast cancer cells to be monitored, which revealed different behavior of each population. In the cancerous mimics, ADSCs adopted an enhanced myofibroblast phenotype, confirmed by increased *α*-SMA expression and a CAF-like secretome, that corroborates the phenotypical changes observed in the matrix model. In addition, these microenvironments containing ADSCs promoted adoption of an invasive cancer stem cell-like state in the breast cancer cells. When breast cancer cells were treated with doxorubicin, the co-occurrence of CAF phenotypes and cancer stem cell-like states in the cancerous mimic lead to increased drug resistance. These results emphasize how structural properties of the matrix influence cancer cell behavior through a combination of mechanical and biochemical cues. While our study did not directly quantify proliferation, it is important to note that variations in matrix compactness and mechanical properties will influence cell division over extended culture periods and across different cancer and stromal cell types.

Integrating this platform into a prototype drug screen demonstrated drug sensitivity corresponding to distinct microenvironments, providing the first platform where matrix, cancer cell state and CAF activity can be controllably assayed in a single experiment. Assaying the secretome from the co-cultures in conjunction provides information on the soluble signals that regulate cancer-stromal cell crosstalk, and opens up avenues for discovering novel biomarkers associated with progression. This platform provides a tool for fundamental discovery and for developing new therapeutic interventions that target specific cancer cell phenotypes as well as tumor–stroma interactions.

## Data Availability

All data that support the findings of this study are included within the article (and any supplementary files).

## References

[1] Mehraj U, Dar A H, Wani N A, Mir M A (2021). Tumor microenvironment promotes breast cancer chemoresistance. Cancer Chemother. Pharmacol..

[2] Terceiro L E L, Edechi C A, Ikeogu N M, Nickel B E, Hombach-Klonisch S, Sharif T, Leygue E, Myal Y (2021). The breast tumor microenvironment: a key player in metastatic spread. Cancers.

[3] Chan Y W, So C, Yau K L, Chiu K C, Wang X, Chan F L, Tsang S Y (2020). Adipose-derived stem cells and cancer cells fuse to generate cancer stem cell-like cells with increased tumorigenicity. J. Cell Physiol..

[4] Rafaeva M (2023). Fibroblast-derived matrix models desmoplastic properties and forms a prognostic signature in cancer progression. Front. Immunol..

[5] Gunaydin G (2021). CAFs interacting with TAMs in tumor microenvironment to enhance tumorigenesis and immune evasion. Front. Oncol..

[6] Koellensperger E, Bonnert L C, Zoernig I, Marmé F, Sandmann S, Germann G, Gramley F, Leimer U (2017). The impact of human adipose tissue-derived stem cells on breast cancer cells: implications for cell-assisted lipotransfers in breast reconstruction. Stem Cell Res. Ther..

[7] Li Z, Wang S, Fang S, Li X, Li Y, Liu G (2022). Adipose-derived stem cells promote the proliferation, migration, and invasion of oral squamous cell carcinoma cells by activating the Wnt/planar cell polarity signaling pathway. Transl. Cancer Res..

[8] Li T, Zhou X, Wang J, Liu Z, Han S, Wan L, Sun X, Chen H (2020). Adipose-derived mesenchymal stem cells and extracellular vesicles confer antitumor activity in preclinical treatment of breast cancer. Pharmacol. Res..

[9] Ko E, Yoon T, Lee Y, Kim J, Park Y B (2023). ADSC secretome constrains NK cell activity by attenuating IL-2-mediated JAK-STAT and AKT signaling pathway via upregulation of CIS and DUSP4. Stem Cell Res. Ther..

[10] Wilson W C, Boland T (2003). Cell and organ printing 1: protein and cell printers. Anatomical Rec. A.

[11] Santoni S, Gugliandolo S G, Sponchioni M, Moscatelli D, Colosimo B M (2021). 3D bioprinting: current status and trends—a guide to the literature and industrial practice. Bio-Design Manuf..

[12] Muir V G, Weintraub S, Dhand A P, Fallahi H, Han L, Burdick J A (2023). Influence of microgel and interstitial matrix compositions on granular hydrogel composite properties. Adv. Sci..

[13] Hinton T J, Jallerat Q, Palchesko R N, Park J H, Grodzicki M S, Shue H-J, Ramadan M H, Hudson A R, Feinberg A W (2015). Three-dimensional printing of complex biological structures by freeform reversible embedding of suspended hydrogels. Sci. Adv..

[14] Highley C B, Song K H, Daly A C, Burdick J A (2019). Jammed microgel inks for 3D printing applications. Adv. Sci..

[15] Skylar-scott M A, Uzel S G M, Nam L L, Ahrens J H, Truby R L, Damaraju S, Lewis J A (2019). Biomanufacturing of organ-specific tissues with high cellular density and embedded vascular channels. Sci. Adv..

[16] Daly A C, Riley L, Segura T, Burdick J A (2020). Hydrogel microparticles for biomedical applications. Nat. Rev. Mater..

[17] Darling N J, Xi W, Sideris E, Anderson A R, Pong C, Carmichael S T, Segura T (2020). Click by click microporous annealed particle (MAP) scaffolds. Adv. Healthcare Mater..

[18] Widener A E, Bhatta M, Angelini T E, Phelps E A (2021). Guest-host interlinked PEG-MAL granular hydrogels as an engineered cellular microenvironment. Biomater. Sci..

[19] Morley C D, Flores C T, Drake J A, Moore G L, Mitchell D A, Angelini T E (2022). Spatiotemporal T cell dynamics in a 3D bioprinted immunotherapy model. Bioprinting.

[20] Reynolds D S (2023). Microporogen-structured collagen matrices for embedded bioprinting of tumor models for immuno-oncology. Adv. Mater..

[21] Zheng X, Hou Y, Zhang Q, Zheng Y, Wu Z, Zhang X, Lin J M (2023). 3D microgel with extensively adjustable stiffness and homogeneous microstructure for metastasis analysis of solid tumor. Chin. Chem. Lett..

[22] Molley T G, Jalandhra G K, Nemec S R, Tiffany A S, Patkunarajah A, Poole K, Harley B A C, Hung T T, Kilian K A (2021). Heterotypic tumor models through freeform printing into photostabilized granular microgels. Biomater. Sci..

[23] Utama R H (2020). A 3D bioprinter specifically designed for the high-throughput production of matrix-embedded multicellular spheroids. iScience.

[24] Jung M S (2022). A high-throughput 3D bioprinted cancer cell migration and invasion model with versatile and broad biological applicability. Biomater. Sci..

[25] Utama R H (2021). A covalently crosslinked ink for multimaterials drop-on-demand 3D bioprinting of 3D cell cultures. Macromol. Biosci..

[26] Molley T G, Hung T T, Kilian K A (2022). Cell-Laden Gradient Microgel suspensions for spatial control of differentiation during biofabrication. Adv. Healthcare Mater..

[27] Romanazzo S, Molley T G, Nemec S, Lin K, Sheikh R, Gooding J J, Wan B, Li Q, Kilian K A, Roohani I (2021). Synthetic bone-like structures through omnidirectional ceramic bioprinting in cell suspensions. Adv. Funct. Mater..

[28] Jalandhra G K, Molley T G, Hung T T, Roohani I, Kilian K A (2023). In situ formation of osteochondral interfaces through ‘bone-ink’ printing in tailored microgel suspensions. Acta Biomater..

[29] San Juan B P (2022). Targeting phenotypic plasticity prevents metastasis and the development of chemotherapy-resistant disease.

[30] Jalandhra G, Romanazzo S, Nemec S, Roohani I, Kilian K A (2022). Ceramic omnidirectional bioprinting in cell-laden suspensions for the generation of bone analogs. J. Vis. Exp..

[31] Wong S L, Kardia E, Vijayan A, Umashankar B, Pandzic E, Zhong L, Jaffe A, Waters S A (2023). Molecular and functional characteristics of airway epithelium under chronic hypoxia. Int. J. Mol. Sci..

[32] Krajina B A, LeSavage B L, Roth J G, Zhu A W, Cai P C, Spakowitz A J, Heilshorn S C (2021). Microrheology reveals simultaneous cell-mediated matrix stiffening and fluidization that underlie breast cancer invasion. Sci. Adv..

[33] Abrahamsson A, Boroojeni F R, Naeimipour S, Reustle N, Selegård R, Aili D, Dabrosin C (2024). Increased matrix stiffness enhances pro-tumorigenic traits in a physiologically relevant breast tissue- monocyte 3D model. Acta Biomater..

[34] Filipe E C (2024). Tumor biomechanics alters metastatic dissemination of triple negative breast cancer via rewiring fatty acid metabolism. Adv. Sci..

[35] Vadhan A, Hou M F, Vijayaraghavan P, Wu Y C, Hu S C S, Wang Y M, Cheng T L, Wang Y Y, Yuan S S F (2022). CD44 promotes breast cancer metastasis through AKT-mediated downregulation of nuclear FOXA2. Biomedicines.

[36] Xu H, Niu M, Yuan X, Wu K, Liu A (2020). CD44 as a tumor biomarker and therapeutic target. Exp. Hematol. Oncol..

[37] Desgrosellier J S, Cheresh D A (2010). Integrins in cancer: biological implications and therapeutic opportunities. Nat. Rev. Cancer.

[38] Li S, Sampson C, Liu C, Piao H L, Liu H X (2023). Integrin signaling in cancer: bidirectional mechanisms and therapeutic opportunities. Cell Commun. Signal..

[39] Levental K R (2009). Matrix crosslinking forces tumor progression by enhancing integrin signaling. Cell.

[40] Engler A J, Sen S, Sweeney H L, Discher D E (2006). Matrix elasticity directs stem cell lineage specification. Cell.

[41] Ping Q, Yan R, Cheng X, Wang W, Zhong Y, Hou Z, Shi Y, Wang C, Li R (2021). Cancer-associated fibroblasts: overview, progress, challenges, and directions. Cancer Gene Ther..

[42] Yang D, Liu J, Qian H, Zhuang Q (2023). Cancer-associated fibroblasts: from basic science to anticancer therapy. Exp. Mol. Med..

[43] Wei H J, Zeng R, Lu J H, Lai W F T, Chen W H, Liu H Y, Chang Y T, Deng W P (2015). Adipose-derived stem cells promote tumor initiation and accelerate tumor growth by interleukin-6 production. Oncotarget.

[44] Promny T, Kutz C S, Jost T, Distel L V, Kadam S, Schmid R, Arkudas A, Horch R E, Kengelbach-Weigand A (2022). An *in vitro* approach for investigating the safety of lipotransfer after breast-conserving therapy. J. Pers. Med..

[45] Zhang M, Zhang B (2025). Extracellular matrix stiffness: mechanisms in tumor progression and therapeutic potential in cancer. Exp. Hematol. Oncol..

[46] Xu R, Yin P, Wei J, Ding Q (2023). The role of matrix stiffness in breast cancer progression: a review. Front. Oncol..

[47] Peng Y, Croce C M (2016). The role of microRNAs in human cancer. Signal Transduct. Target. Ther..

[48] Yang Z, Liu Z (2020). The emerging role of MicroRNAs in breast cancer. J. Oncol..

[49] Yang F, Ning Z, Ma L, Liu W, Shao C, Shu Y, Shen H (2017). Exosomal miRNAs and miRNA dysregulation in cancer-associated fibroblasts. Mol. Cancer.

[50] Tang F, Zhang R, He Y, Zou M, Guo L, Xi T (2012). MicroRNA-125b induces metastasis by targeting STARD13 in MCF-7 and MDA-MB-231 breast cancer cells. PLoS One.

[51] Wang D, Sang Y, Sun T, Kong P, Zhang L, Dai Y, Cao Y, Tao Z, Liu W (2021). Emerging roles and mechanisms of microrna 222 3p in human cancer (review). Int. J. Oncol..

[52] Dai H, Xu L Y, Qian Q, Zhu Q W, Chen W X (2019). MicroRNA-222 promotes drug resistance to doxorubicin in breast cancer via regulation of miR-222/bim pathway. Biosci. Rep..

[53] Gao W, Hua J, Jia Z, Ding J, Han Z, Dong Y, Lin Q, Yao Y (2018). Expression of miR-146a-5p in breast cancer and its role in proliferation of breast cancer cells. Oncol. Lett..

[54] Nedaeinia R, Najafgholian S, Salehi R, Goli M, Ranjbar M, Nickho H, Haghjooy Javanmard S, Ferns A G, Manian M (2024). The role of cancer-associated fibroblasts and exosomal miRNAs-mediated intercellular communication in the tumor microenvironment and the biology of carcinogenesis: a systematic review. Cell Death Discovery.

[55] Nath S, Mukherjee P (2014). MUC1: a multifaceted oncoprotein with a key role in cancer progression. Trends Mol. Med..

[56] Mitra P, Yang R-M, Sutton J, Ramsay R G, Gonda T J (2016). CDK9 inhibitors selectively target estrogen receptor-positive breast cancer cells through combined inhibition of MYB and MCL-1 expression. Oncotarget.

[57] Cheng S, Yang G J, Wang W, Ma D L, Leung C H (2022). Discovery of a tetrahydroisoquinoline-based CDK9-cyclin T1 protein–protein interaction inhibitor as an anti-proliferative and anti-migration agent against triple-negative breast cancer cells. Genes Dis..

